# Screw Insertion Time, Fluoroscopy Time, and Operation Time for Robotic-Assisted Lumbar Pedicle Screw Placement Compared With Freehand Technique

**DOI:** 10.7759/cureus.25039

**Published:** 2022-05-16

**Authors:** Yoshiaki Torii, Jun Ueno, Tasuku Umehara, Masahiro Iinuma, Atsuhiro Yoshida, Ken Tomochika, Hisateru Niki, Tsutomu Akazawa

**Affiliations:** 1 Department of Orthopaedic Surgery, St. Marianna University School of Medicine, Kawasaki, JPN; 2 Spine Center, St. Marianna University Hospital, Kawasaki, JPN

**Keywords:** robotic-assisted pedicle screw placement, spinal navigation, screw insertion time, radiation exposure, fluoroscopy, spine robotic system, freehand technique, lumbar spine, pedicle screw placement, robotic-assisted spine surgery

## Abstract

Introduction

The purpose of this study was to clarify the superiority of robotic-assisted lumbar pedicle screw placement in terms of screw insertion time, fluoroscopy time, and operation time.

Methods

The subjects were 46 patients who underwent a posterior lumbar interbody fusion with an open procedure for lumbar degenerative disease from April 2021 to February 2022. The robot group contained 29 cases of screw insertion using a spine robotic system (Mazor X Stealth Edition, Medtronic Inc., Dublin, Ireland). The freehand group contained 17 cases of screw insertion with the freehand technique utilizing the conventional C-arm image guidance. The screw insertion time, fluoroscopy time, and operation time were compared between the robot and the freehand group.

Results

The screw insertion time did not differ significantly between the two groups (robot group: 179.0 ± 65.2 sec; freehand group: 164.2 ± 83.4 sec; p = 0.507). The fluoroscopy time was significantly shorter in the robot group (robot group: 28.3 ± 25.8 sec; freehand group: 67.5 ± 72.8 sec; p = 0.011). The fluoroscopy time per segment was also significantly shorter in the robot group (robot group: 17.8 ± 23.0 sec; freehand group: 60.2 ± 74.8 sec; p = 0.007). The operation time was significantly longer in the robot group (robot group: 249.6 ± 72.5 min; freehand group: 195.8 ± 60.1 sec; p = 0.013), but the operation time per segment did not differ significantly between the two groups (robot group: 144.1 ± 39.0 min; freehand group: 159.7 ± 34.4 min; p = 0.477).

Conclusions

The screw insertion time and operation time per segment were similar when employing the spine robotic system compared to the freehand technique; however, the fluoroscopy time was shorter. The fluoroscopy time per segment in the robot group was 29.6% of the time of the freehand group using the C-arm. The surgeon's radiation exposure is thought to be decreased since the spine robotic system shortens the fluoroscopy time.

## Introduction

Due to the significant rate of deviation recorded with freehand pedicle screw placement without image guidance, many institutions have employed C-arm imaging guidance or spinal navigation. The first spine surgical robotic system, SpineAssist (Mazor Robotics, Caesarea, Israel), was developed in Israel in the early 2000s to increase accuracy and was authorized by the US Food and Drug Administration in 2004 [1]. The Mazor X Stealth Edition, which incorporates a contemporary spinal navigation system, was developed by Medtronic in 2018. According to a meta-analysis, spine robotic systems have a pedicle screw accuracy of 85-100% [2]. The deviation rate of the spine robotic system was minimal when compared to the freehand technique, according to previous meta-analyses [3,4]. The new robotic system with integrated spinal navigation has shown quicker surgery times, shorter fluoroscopy times, and reduced complications, in addition to screw accuracy [5].

Previous reports for robotic-assisted spine surgery focused on the times such as the screw insertion time and operation time. The screw insertion time was previously calculated by dividing the total time by the number of screws in prior reports. The screw insertion time was not measured for each screw.

The purpose of this study was to clarify the superiority of robotic-assisted lumbar pedicle screw placement in terms of screw insertion time, fluoroscopy time, and operation time. Regarding the screw insertion time, the insertion time was accurately measured for each screw.

## Materials and methods

Study subjects

The Institutional Review Board of St. Marianna University School of Medicine gave their approval to this retrospective study (approval code: 5478). The subjects were 46 patients who underwent a posterior lumbar interbody fusion with an open procedure for lumbar degenerative diseases from April 2021 to February 2022. This study included lumbar spinal stenosis cases without deformity. This study excluded patients who underwent posterior lateral fusion, percutaneous pedicle screw fixation, or thoracic spine fusion. The subjects were divided into two groups: the robot group and the freehand group. The robot group contained 29 cases of screw insertion using a spine robotic system (Mazor X Stealth Edition, Medtronic Inc., Dublin, Ireland). The freehand group contained 17 cases of screw insertion with the freehand technique utilizing the conventional C-arm image guidance. Five surgeons conducted pedicle screw insertions, and they have a combined experience of 5, 7, 10, 17, and 20 years as spine surgeons. Before this investigation, the surgeons had no experience with robotic-assisted screw placement. The selection of the group was based on the use of a spine robotic system. Screw insertion was conducted utilizing the freehand technique in the C-arm guidance when the spine robotic system could not be employed due to the surgery being performed on the same day. When choosing surgery, we basically prioritized robotic-assisted surgery. The shape of the facets or the other degenerative changes did not change the selection. The screw insertion time, fluoroscopy time, and operation time were compared between the robot and the freehand group.

Time's definition

Screw Insertion Time

The insertion time was recorded for each pedicle screw. The insertion time was calculated as the time between drilling a pilot hole and installing screws. The screw insertion time was used to calculate the mean insertion time in each case.

Fluoroscopy Time

The fluoroscopy time was calculated by subtracting the cage placement time from the total fluoroscopy time during surgery. The C-arm was used when placing the cage, but the fluoroscopy time did not include the time spent on discectomy or cage insertion.

Fluoroscopy Time per Segment

The fluoroscopy time per segment was calculated by dividing fluoroscopy time by the number of fusion segments. The C-arm was used when placing the cage, but the fluoroscopy time per segment did not include the time spent on discectomy or cage insertion.

Operation Time

The time from skin incision to closure was defined as the operation time. This included all procedures such as approach, registration time, implant placement, and decompression.

Operation Time per Segment

The operation time per segment was calculated by dividing the operation time by the number of fusion segments.

Operative procedure

A skin incision was created in the targeted fusion area's posterior midline. All the procedures were done through a posterior approach, using midline fascial incisions or midline skin and separate fascial Wiltse incisions.

**Figure 1:**
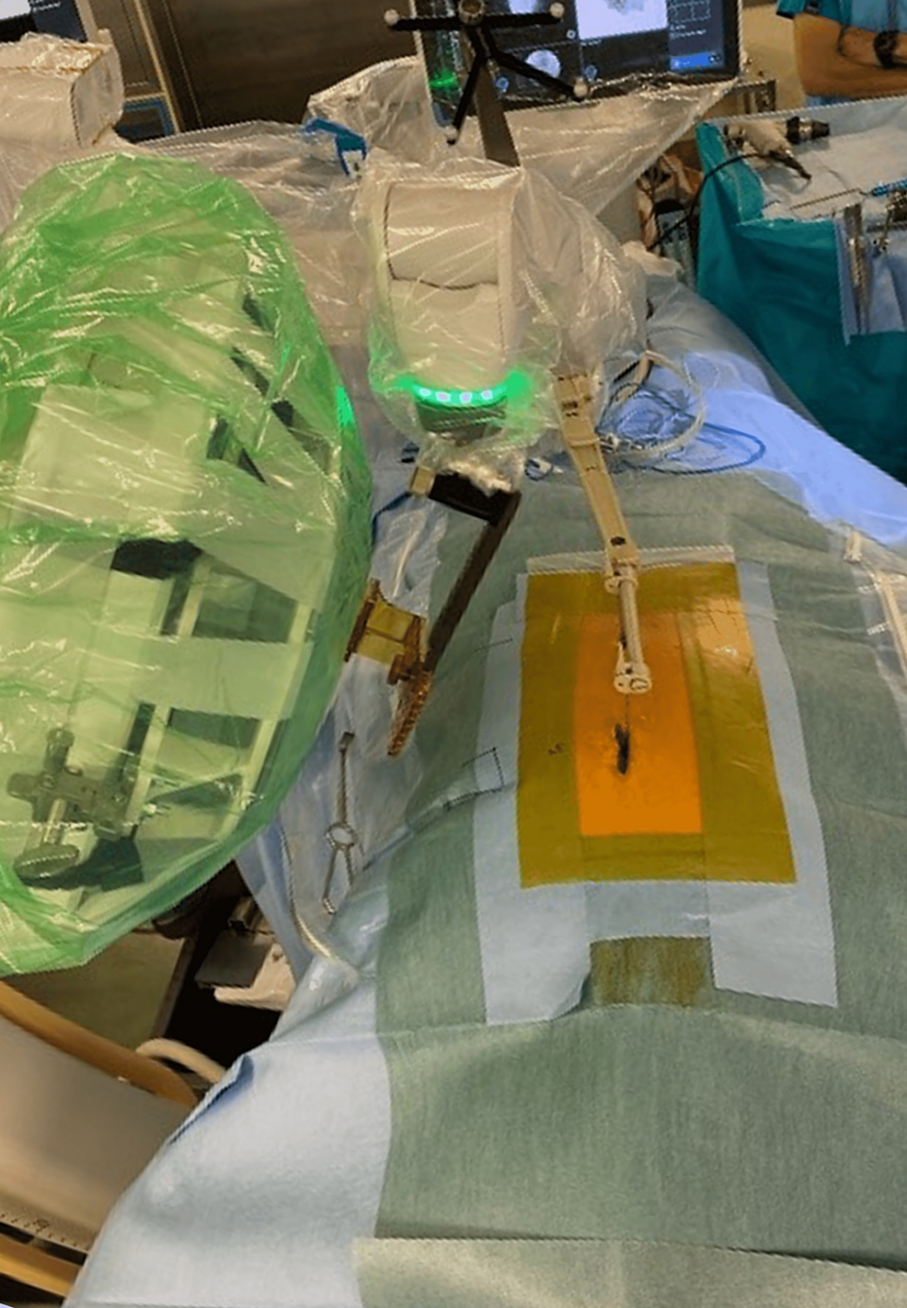
CT to fluoro workflow The C-arm was used to acquire frontal and oblique X-ray images during surgery in the robot group, which were matched with the planning data.

**Figure 2:**
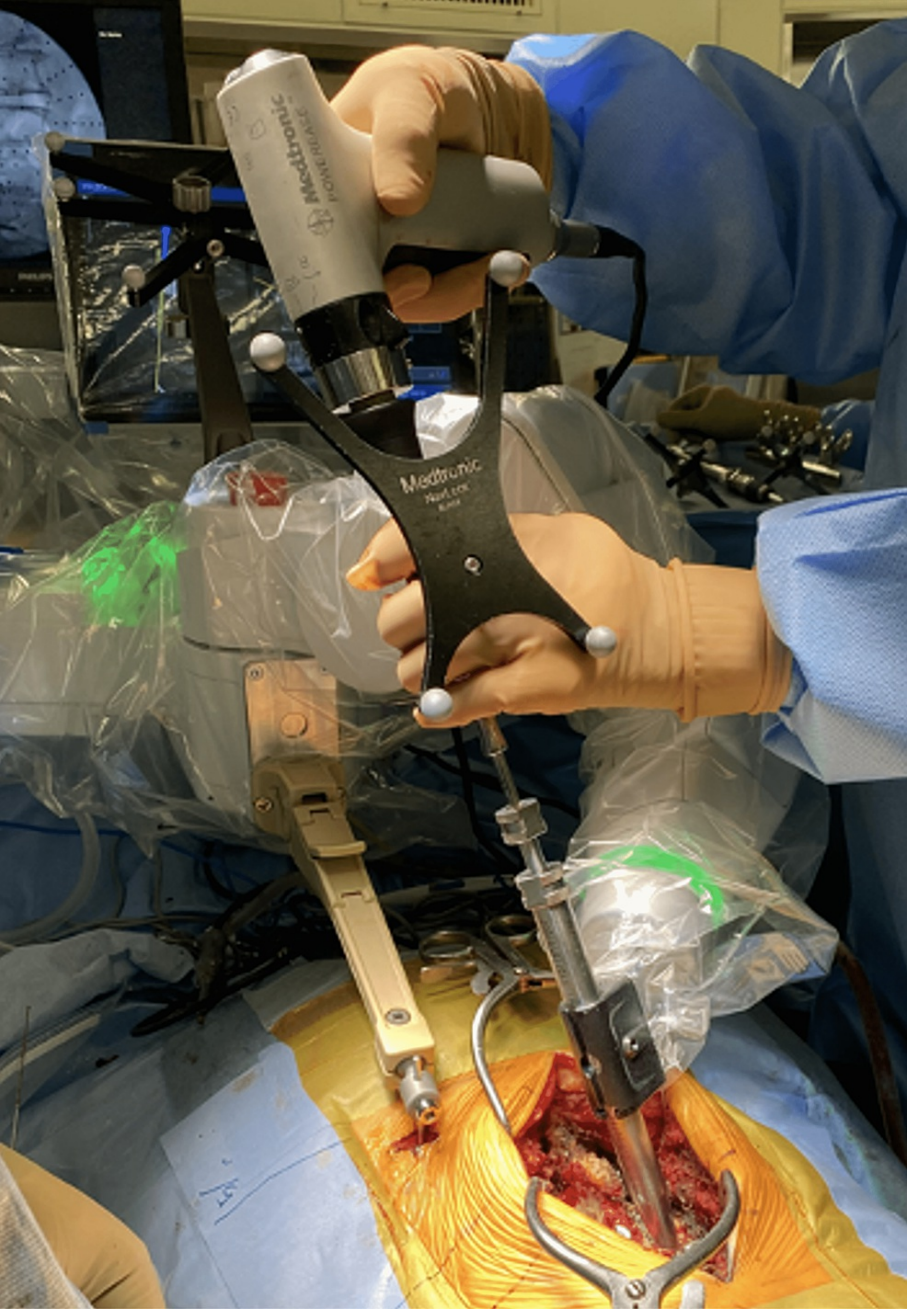
Robotic-assisted pedicle screw placement In the robot group, pedicle screws were inserted under the robotic arm guide without Kirschner-wire guidance.

In the robot group, a spine robotic system was used to place the lumbar pedicle screws. We performed all surgeries using the “CT to fluoro” workflow. Preoperative CT images were obtained, and the preoperative CT images were used to plan pedicle screw placements. The spine robotic system was programmed with the planning data. The C-arm (STX-1000A, Toshiba Medical Systems, Otawara, Japan or Zenition 70, Philips, Amsterdam, Netherlands) was used to acquire frontal and oblique X-ray images during surgery, which were matched with the planning data (Figure 1). Without Kirschner-wire guidance, pedicle screws were inserted under the robotic arm guide (Figure 2). After placing all the screws, we confirmed the proper position on the frontal and lateral views.

In the freehand group, pedicle screws were placed with the freehand technique under the guidance of the C-arm. We made the pilot holes, probed, tapped, and placed screws in the lateral view. After placing all the screws, we confirmed the proper position on the frontal view.

After the intervertebral disc was removed, bone grafting and cage implantation were performed for interbody fusion at all the fusion segments, and the wound was closed.

Statistical analysis

A Student’s t-test and Fisher's exact test with chi-square were used as the statistical analyses. Normally distributed continuous variables were expressed as mean ± standard deviation. Significant differences were defined as p < 0.05.

## Results

**Figure 3:**
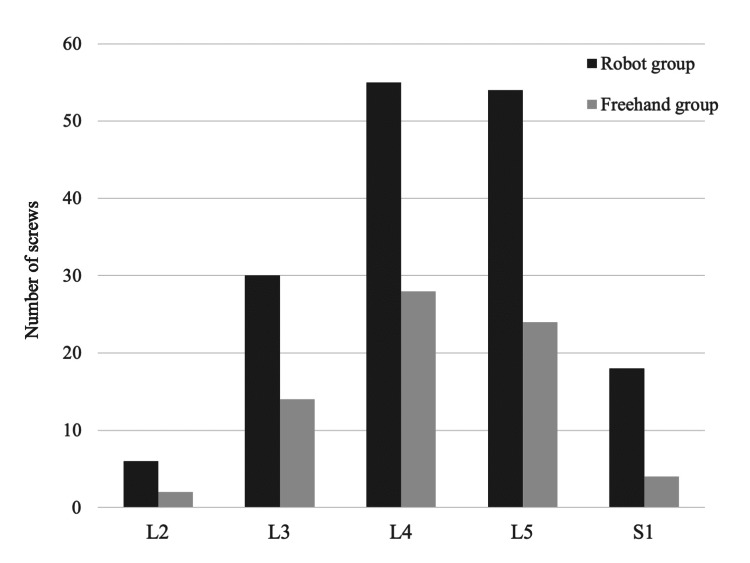
The number of pedicle screws at each vertebral body

There was no significant difference between the two groups in age, gender, or body mass index. The number of fusion segments was larger in the robot group than those in the freehand group (Table 1). The number of pedicle screws at each vertebral body is shown in Figure 3. There was no significant difference in the vertebral levels between the two groups (p = 0.712).

**Table 1:** Demographic data Variables were expressed as mean ± standard deviation.

	Robot group	Freehand group	P-value
Age	69.7 ± 11.7	72.0 ± 12.1	0.536
Gender			1.000
Female	16	9	
Male	13	8	
Body mass index	24.2 ± 4.4	23.6 ± 4.1	0.626
Number of fusion segments			0.025
1 segment	10	13	
2 segments	14	3	
3 segments	5	1	

The screw insertion time did not differ significantly between the two groups (robot group: 179.0 ± 65.2 sec; freehand group: 164.2 ± 83.4 sec; p = 0.507). The fluoroscopy time was significantly shorter in the robot group (robot group: 28.3 ± 25.8 sec; freehand group: 67.5 ± 72.8 sec; p = 0.011). The fluoroscopy time per segment was also significantly shorter in the robot group (robot group: 17.8 ± 23.0 sec; freehand group: 60.2 ± 74.8 sec; p = 0.007). The operation time was significantly longer in the robot group (robot group: 249.6 ± 72.5 min; freehand group: 195.8 ± 60.1 min; p = 0.013), but the operation time per segment did not differ significantly between the two groups (robot group: 144.1 ± 39.0 min; freehand group: 159.7 ± 34.4 min, p = 0.477) (Table 2).

**Table 2:** The comparison of screw insertion time, operation time, and fluoroscopy time in the robot group and the freehand group Variables were expressed as mean ± standard deviation.

	Robot group	Freehand group	P-value
Screw insertion time (sec)	179.0 ± 65.2	164.2 ± 83.4	0.507
Fluoroscopy time (sec)	28.3 ± 25.8	67.5 ± 72.8	0.011
Fluoroscopy time per segment (sec)	17.8 ± 23.0	60.2 ± 74.8	0.007
Operation time (min)	249.6 ± 72.5	195.8 ± 60.1	0.013
Operation time per segment (min)	144.1 ± 39.0	159.7 ± 34.4	0.477

## Discussion

In terms of screw insertion time, there was no significant difference between the robot and freehand groups. The operation time per segment did not differ significantly between the two groups; however, the fluoroscopy time per segment was significantly shorter in the robot group. The screw insertion and operation times were comparable to those of the freehand technique; however, the fluoroscopy time was shorter when a spine robotic system was used. The fluoroscopy time per segment in the robot group was 29.6% of the time of the freehand group using the C-arm. The spine robotic system shortened the fluoroscopy time, which might contribute to the reduction of radiation exposure.

Screw insertion time decreased with surgeons’ experience in robot-assisted pedicle screw placement, according to several research studies [6-9]. According to Bäcker et al., the average insertion time per pedicle screw was 8.6 minutes, with a decreasing operating time trend [8]. Hyun et al. stated that the average insertion time per screw was lowered from 5.5 minutes in the first 15 cases to 4.0 minutes in the next 15 cases [7]. In this study, the screw insertion time did not differ significantly between the robot and freehand groups. This study included early cases of robotic-assisted spine surgery. As the surgeons become more proficient in robotic-assisted spine surgery, spine robotic systems may have a shorter screw insertion time than the freehand technique.

In a meta-analysis, it was reported that the robotic-assisted group had better screw accuracy than the freehand group, but the skin-to-skin operation time was longer [4]. The increase in time during surgery may be due to the learning curve by the robot and the time required to acquire and register the images during surgery for navigation [3]. In this study, the robot group had a long overall operation time, which is thought to be due to a large number of fusion segments. The operation time per segment was similar between the robot group and the freehand group. The screw insertion time was also similar in the two groups. These findings suggested that robotic-assisted spine surgery was comparable to conventional freehand surgery in terms of time. The learning curve for robotic-assisted spine surgery has been reported in many ways, ranging from no learning curve to 30 cases necessary to achieve proficiency [8-11]. This study showed that the screw insertion time was favorable in the robot group even in the cases of the early phase. The screw insertion time and operation time might become shorter with experience.

Radiation exposure during surgery has been linked to an increased risk of cancer in surgeons and operating room workers [12-16]. As a result, limiting radiation exposure during surgery saves the surgeon's health. According to a meta-analysis, patients who had robot-assisted spine surgery had much-reduced radiation times and radiation exposure than those who had freehand technique [4]. Although the radiation exposure was not directly measured in this study, the total fluoroscopy time and the fluoroscopy time per segment were both shorter in the robot group than in the freehand group. We have shown that robot-assisted spine surgery can reduce reliance on intraoperative fluoroscopy.

There were several limitations in this study. Since this was a retrospective study, the number of cases and the number of fusion segments in the robot and freehand groups were not equivalent. Since the robot group had more fusion segments, the total operation time was longer for the robot group than for the freehand group. However, the operation time per segment was the same in both groups, so it did not mean that robotic-assisted spine surgery would take longer. This was not a randomized controlled trial. The availability of the spine robotic system determined whether robotic-assisted screw placement or conventional freehand technique was performed. As a result, the probability of selection bias could not be discounted. In this study, the radiation dose was not measured. Although the exposure dose was not measured directly, the fluoroscopy time was significantly shorter in the robot group, so it is inferred that the use of the robot reduced the radiation exposure to the surgeons. The fluoroscopy time might decrease with experience, but this was not considered in this study.

## Conclusions

The screw insertion time and the operation time per segment were similar when employing the spine robotic system compared to the freehand technique; however, the fluoroscopy time was shorter in the robot group. The fluoroscopy time per segment in the robot group was 29.6% of the time of the freehand group using the C-arm. The surgeon's radiation exposure is thought to be decreased since the spine robotic system shortens the fluoroscopy time.
